# A case report of idiopathic pleuroparenchymal fibroelastosis with severe respiratory failure in pregnancy

**DOI:** 10.1186/s12890-020-01308-2

**Published:** 2020-10-14

**Authors:** Aiko Sekine, Kohei Seo, Satoshi Matsukura, Masaaki Sato, Aya Shinozaki-Ushiku, Takashi Ogura, Akihiko Kitami, Mitsutaka Kadokura, Satoshi Dohi, Kiyotake Ichizuka, Masaaki Nagatsuka

**Affiliations:** 1 Department of Obstetrics and Gynecology, Showa University Northern Yokohama Hospital, Chigasaki-chuo 35-1, Tsuzuki- ku, Yokohama-shi, Kanagawa, 224-8503 Japan; 2 Respiratory Disease Center, Showa University Northern Yokohama Hospital, Chigasaki-chuo 35-1, Tsuzuki-ku, Yokohama-shi, Kanagawa, 224-8503 Japan; 3 Department of Thoracic Surgery, University of Tokyo Graduate School of Medicine, Hongo 7-3-1, Bunkyo-ku, Tokyo, 113-0033 Japan; 4 Department of Pathology, University of Tokyo Graduate School of Medicine, Hongo 7-3-1, Bunkyo-ku, Tokyo, 113-0033 Japan; 5 Department of Respiratory Medicine, Kanagawa Cardiovascular and Respiratory Center, Tomiokahigasi 6-16-1, Kanazawa-ku, Yokohama-shi, Kanagawa, Japan

**Keywords:** Acute exacerbation, Idiopathic pleuroparenchymal fibroelastosis, Lung transplant, Pregnancy, Case report

## Abstract

**Background:**

Idiopathic pleuroparenchymal fibroelastosis (IPPFE) is a rare lung disease that manifests as parenchymal fibrosis of the upper lung lobe and pleura. There have been no reports of IPPFE complicating pregnancy. Here, we report a case of IPPFE that deteriorated rapidly during pregnancy.

**Case presentation:**

A 29-year-old woman presented with dyspnea and dry cough at 19 weeks of gestation. IPPFE with acute exacerbation was suspected on chest computed tomography (CT). Despite steroid treatment, her condition progressed. A cesarean section was performed at 28 weeks of gestation. On postoperative day 26, she underwent living-donor lung transplantation. She was discharged a year after transplantation.

**Conclusion:**

Our experience suggested that when pregnancy is complicated by PPFE, the disease may deteriorate rapidly. In this case, even though IPPFE with acute exacerbation was diagnosed during pregnancy, live birth was achieved, and the mother survived after lung transplantation. Lung transplantation should be considered in these patients because, once advanced, pulmonary lesions may be irreversible.

## Background

Idiopathic pleuroparenchymal fibroelastosis (IPPFE) is a rare entity characterized by upper lobe-dominant subpleural fibroelastosis and dense fibrous thickening of the visceral pleura [1]. According to some reports, this disease progresses slowly; however, there have been reports of rapid deterioration [2–4]. It has been reported that the onset age is highly heterogeneous, ranging from 13 to 87 years, and bimodal peaks occur in the 30 s and 60 s [5]. There are no effective medications, and the prognosis is considered poor; therefore, diagnosis and careful observation during the asymptomatic stage are important, and early lung transplantation should be considered when symptoms appear.

No cases of IPPFE complicating pregnancy have been reported. Here, we report our experience of a young woman with IPPFE that deteriorated rapidly during pregnancy. In this case, live birth was achieved, and the mother was subsequently able to undergo lung transplantation.

## Case presentation

The patient was a primigravida, para 0, 29-year-old woman with unremarkable family history and a medical history of idiopathic thrombocytopenia. Her chief complaints were cough and respiratory distress.

**Fig. 1:**
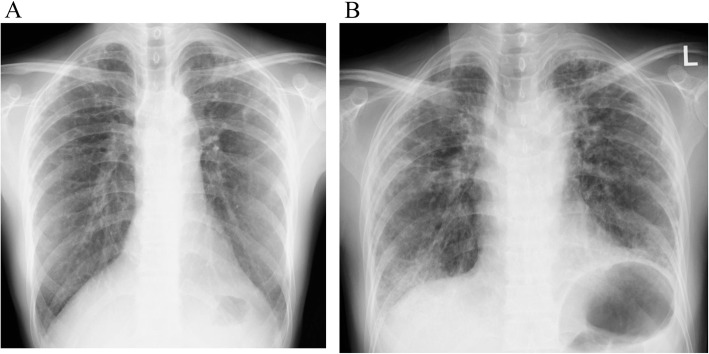
Chest radiography findings. (**a**) Chest radiography 3 years before admission showed apical pleural thickening and small reticular opacity (**b**) Chest radiography at 24 weeks of gestation showed subpleural thickening, reduced pneumatization in the upper lung, and reticular opacity throughout the lung

**Fig. 2:**
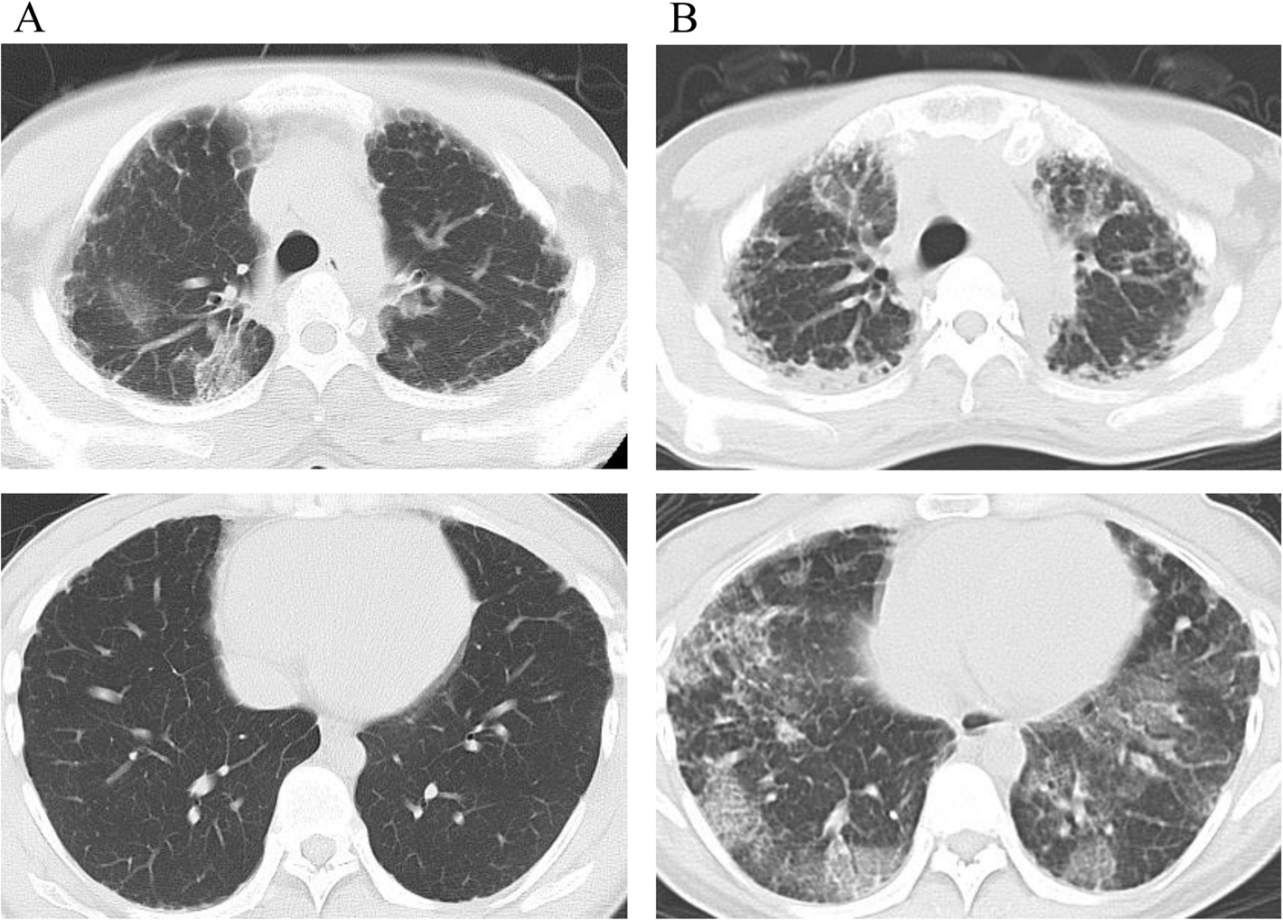
Chest computed tomography findings (CT). (**a**) Chest CT 3 years before admission showed apical pleural thickening and reticular opacity in the peripheral lung field (**b**) Chest CT at 24 weeks of gestation showed upper lobe-predominant pleural thickening, bronchiectasis, reticular opacity, and ground-glass opacity throughout the lung

The patient had been examined 3 years previously for cough. Chest radiography and computed tomography (CT) showed bilateral apical pleural thickening and reticular opacity in the peripheral lung field (Figs. 1 and 2), but no further examinations were performed. At 19 weeks of gestation, she was examined at our hospital for cough and respiratory distress. In room air, she exhibited an SpO_2_ of 97% and good oxygenation. At 25 weeks of gestation, she was examined again due to worsening cough and respiratory distress. Her SpO_2_ had declined to 92% in room air, and she was admitted to the hospital.

On admission, the patient’s height was 164 cm, weight was 47.8 kg, body temperature was 37.0 °C, PaO_2_ was 68.1 mmHg, and PaCO_2_ was 26.5 mmHg in room air. Fine crackles could be heard on the dorsal side of the lung; she had clubbed fingers.

Hematological examination on admission revealed a white blood cell count of 5750/μL, hemoglobin level of 9.5 g/dL, platelet level of 67,000/μL, C-reactive protein level of 3.75 mg/d/l, Krebs von den Lungen-6 level of 945 U/mL (reference interval: < 500), and serum surfactant protein D of 274.5 ng/mL (reference interval: < 110). She tested negative for any autoantibodies and infectious diseases.

Chest radiography revealed bilateral pleural thickening, reticular opacity, and reduced pneumatization predominantly in the upper lung (Fig. 1). Chest CT showed bronchiectasis and irregular pleural thickening mainly in the bilateral upper lobes, reticular opacity, and ground-glass opacity (GGO) throughout the lung (Fig. 2). Image findings worsened compared to 3 years ago. Abdominal ultrasonography estimated the fetal body weight at 886 g (+ 0.2 standard deviation) and revealed an appropriate amount of amniotic fluid and active fetal movement.

The image findings, subpleural dense consolidation, bronchodilation, and reduced pneumatization, predominantly in the bilateral upper lobes, were characteristic of PPFE. Other differential diseases were excluded on account of the clinical course, blood tests, and bacteriological tests. As a result of a conference between radiologists and respiratory physicians with reference to the literature [1, 6], the patient was diagnosed with probable IPPFE with acute exacerbation. Prednisolone 30 mg/day was started at 26 weeks and 1 day of gestation, but no changes were observed; thus, methylprednisolone 1,000 mg/day was administered for 3 days starting at 27 weeks and 3 days’ gestation. Left pneumothorax occurred at 27 weeks and 5 days’ gestation. Fetal status was evaluated daily using a fetal heart rate monitor and Doppler examinations, and weekly using ultrasonography. Throughout the course, the well-being of the fetus was good, and growth was normal. At 27 weeks and 6 days’ gestation, the patient’s respiratory status had worsened; with 4 L of oxygen, SpO_2_ was 94%, PaO_2_ was 67.9 mmHg, and PaCO_2_ was 29.3 mmHg. Chest CT findings also showed a worsening trend; therefore, steroid therapy was considered ineffective.

To improve the mother’s respiratory status and prioritize the treatment of IPPFE, a cesarean section was performed at 28 weeks and 0 days’ gestation. Preoperatively, a transfusion of 10 units of platelets was administered. Spinal anesthesia was administered. The operation took 46 min, and there was 848 g of blood loss. The baby was a boy weighing 1,185 g, with an Apgar score of 7 and 9 at 1 min and 5 min, respectively, and an umbilical artery pH of 7.320. He was transferred to the neonatal intensive care unit.

**Fig. 3:**
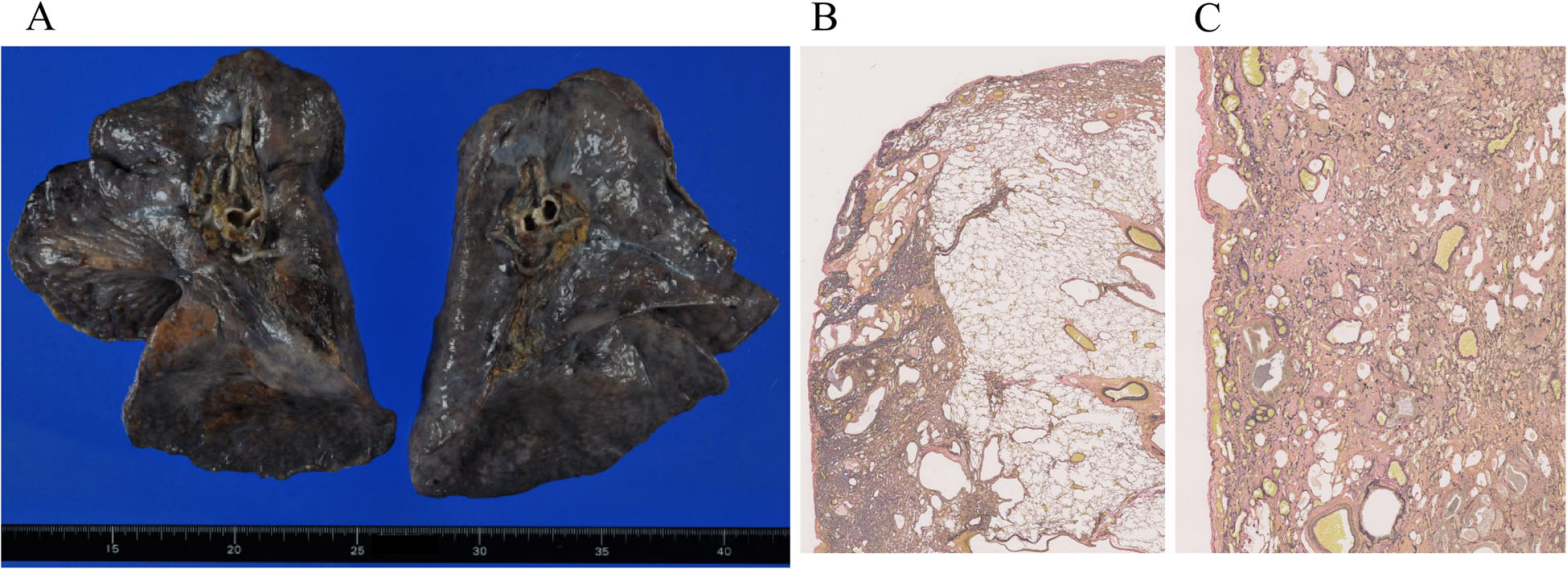
Descriptions of the lobes. (**a**) Gross description: upper lobes were reduced and pleura was irregularly thickened (**b**) Microscopic description of the upper lobe: aggregations of alveolar elastic fibers and collagen fiber growth; Van Gieson elastic staining (**c**) Microscopic description of the lower lobe: extensive organization; Van Gieson elastic staining

Immediately after the operation, SpO_2_ improved to 98% with 3 L of oxygen, and the patient’s respiratory distress decreased. However, respiratory distress worsened again on postoperative day 8, and 5 L or more of oxygen was needed to keep SpO_2_ from falling below 90%. Chest CT indicated bilateral worsening of the GGO. Methylprednisolone 1,000 mg/day was administered for 3 days, after which immunosuppressant therapy was co-administered with prednisolone 30 mg/day and cyclosporine 3 mg/kg/day. On postoperative day 22, the patient was transferred to another hospital for living-donor lung transplantation. On postoperative day 26, she underwent bilateral living-donor lung transplantation from her mother and brother. The extracted lung showed aggregations of alveolar elastic fibers predominantly in the upper lobes and collagen fiber growth that appeared to fill the alveoli (Fig. 3). These findings were compatible with those of IPPFE. In addition, the organization was widely distributed in the lower lobes.

Although her recovery immediately after lung transplantation was relatively uneventful, her clinical course after a month was eventful including repeated nausea and vomiting for unknown reasons which eventually necessitated gastrostomy and jejunostomy; multiple episodes of critical acute rejection, one of which necessitated extracorporeal membrane oxygenation support; and multiple episodes of infection including repeated pneumonia and empyema. She was finally discharged a year after transplantation. Although she still has relatively minor episodes of rejection and infection necessitating temporary admission, she has become relatively stable nearly 2 years after lung transplantation. Hematologically, her relatively mild pancytopenia persisted after lung transplantation. Platelet transfusion was repeatedly needed just after lung transplantation; subsequently, granulocyte colony-stimulating factor administration has been repeatedly needed.

The baby weighed 3,390 g at age 84 days (corrected: 40 weeks 0 days) and did not display any neurological abnormalities.

## Discussion and conclusion

The median survival period of PPFE has been reported to be 7.3 years [2] and 11 years [3]. According to some reports, this disease progresses slowly; however, there have been reports of rapid deterioration [2–4]. Therapy with steroids, pirfenidone, immunosuppressants, and N-acetylcysteine has been reported without marked effects. Lung transplants have been reported [7–11], and one study reported that the patient was alive at 5 years with a good quality of life. However, there have been no reports of IPPFE complicating pregnancy.

Symptoms in the present case appeared when the patient was 19 weeks pregnant. Over the next 6 weeks, her SpO_2_ and PaO_2_ declined and image findings worsened, suggesting that pregnancy may have been the cause for the rapid deterioration of the patient’s condition. We speculate that the effects of pregnancy-related changes on respiratory function could have caused the rapid worsening of PPFE. During pregnancy, the tidal volume and oxygen consumption increase. Therefore, it is possible that, due to the restrictive impairments of IPPFE, the patient could not respond to such changes, leading to the rapid deterioration of her condition. However, in reports of cases of pulmonary restrictive impairment during pregnancy, the disease worsened in some cases to the extent that pregnancy had to be terminated, while no changes were observed in other cases [12, 13]. One retrospective study reported that connective tissue disease-associated interstitial lung disease was not associated with rapid worsening [14]; however, it is unclear whether the same applies to patients who do not have connective tissue disease, as in this case.

Maternal hypoxemia increases the risk of miscarriage, premature birth, and intrauterine growth retardation, and maternal oxygen saturation reportedly correlates with the fetal survival rate. Presbitero et al. observed fetal survival rates of 12%, 45%, and 92% when SpO_2_ was ≤ 85%, 85 − 89%, and ≥ 90%, respectively [15]. We planned to deliver the fetus to save its life if SpO_2_ could not be maintained at ≥ 90%. It was also decided that the pregnancy would be terminated to save the mother’s life, if doing so could be expected to improve her respiratory status or if pregnancy placed limitations on necessary treatment. The mother did not respond to pharmacotherapy, and her subjective symptoms, physical findings, and image features worsened. Moreover, at 28 weeks of gestation, it was thought that the infant could survive outside the womb and, thus, the pregnancy was terminated to save the mother’s life. However, after delivery, the pulmonary lesions continued to progress. This suggests that in cases of PPFE during pregnancy, terminating the pregnancy will not necessarily improve the progression of the primary disease.

In the present case, even though IPPFE was diagnosed during pregnancy and the disease progressed rapidly, live birth was achieved, and the mother survived after lung transplantation. IPPFE could progress during pregnancy before the fetus can mature which could put both the mother and child at risk of death. Close teamwork between the obstetrics, respiratory, neonatal, and other departments is therefore important in such cases.

In conclusion, our experience suggests that when pregnancy is complicated by PPFE, the disease may deteriorate rapidly, although the underlying mechanism is unclear, and termination of the pregnancy cannot be expected to lead to an improvement in PPFE. Lung transplantation should be considered in these patients because, once advanced, pulmonary lesions may be irreversible.

## Data Availability

The datasets used in this study are available from the corresponding author on reasonable request.

## Abbreviations

IPPFE: Idiopathic pleuroparenchymal fibroelastosis
CT: Computed tomography
GGO: Ground-glass opacity

## References

[1] Travis WD, Costabel U, Hansell DM, King TE, Lynch DA, Nicholson AG (2013). An official American Thoracic Society/European Respiratory Society statement: update of the international multidisciplinary classification of the idiopathic interstitial pneumonias. Am J Respir Crit Care Med.

[2] Yoshida Y, Nagata N, Tsuruta N, Kitasato Y, Wakamatsu K, Yoshimi M (2016). Heterogeneous clinical features in patients with pulmonary fibrosis showing histology of pleuroparenchymal fibroelastosis. Respir Investig.

[3] Watanabe K (2013). Pleuroparenchymal fibroelastosis: its clinical characteristics. Curr Respir Med Rev.

[4] Oda T, Ogura T, Kitamura H, Hagiwara T, Baba T, Enomoto T (2014). Distinct characteristics of pleuroparenchymal fibroelastosis with usual interstitial pneumonia compared with idiopathic pulmonary fibrosis. Chest.

[5] Bonifazi M, Montero MA, Renzoni EA (2017). Renzoni Idiopathic pleuroparenchymalfibroelastosis. CurrPulmonol Rep.

[6] Reddy TL, Tominaga M, Hansell DM, von der Thusen J, Rassl D, Parfley H (2012). Pleuroparenchymal fibroelastosis: a spectrum of histopathological and imaging phenotypes. Eur Respir J.

[7] Chen F, Matsubara K, Miyagawa-Hayashino A, Tada K, Handa T, Yamada T (2014). Lung transplantation for pleuroparenchymal fibroelastosis after chemotherapy. Ann Thorac Surg.

[8] Hata A, Nakajima T, Yoshida S, Kinoshita T, Terada J, Tatsumi K (2016). Living donor lung transplantation for pleuroparenchymal fibro elastosis. Ann Thorac Surg.

[9] Yanagiya M, Sato M, Kawashima S, Kuwano H, Nagayama K, Nitadori J (2016). Flat chest of pleuroparenchymal fibroelastosis reversed by lung transplantation. Ann Thorac Surg.

[10] Huang H, Feng R, Li S, Wu B, Xu K, Xu Z (2017). A CARE-compliant case report: lung transplantation for a Chinese young man with idiopathic pleuroparenchymal fibroelastosis. Medicine (Baltimore).

[11] Righi I, Morlacchi L, Rossetti V, Mendogni P, Palleschi A, Tosi D (2019). Lung transplantation as successful treatment pf end-stage idiopathic pleuroparencymal fibroelastosis. Transplant Proc.

[12] Lapinsky SE, Tram C, Mehta S, Maxwell CV (2014). Restrictive lung disease in pregnancy. Chest.

[13] Ohira S, Asaka R, Tanaka Y, Fuseya C, Ando H, Kkuchi N (2017). Management of a pregnant woman with idiopathic interstitial pneumonia accompanied by secondary pulmonary hypertension: case report and literature review. Med Case Rep.

[14] Giattino SL, Eudy AM, Clowse MEB. Pregnancy outcomes in patients with interstitial lung disease related to autoimmune disease and sarcoidosis. Arthritis Rheumatol. 2018;70 (suppl 10)..

[15] Presbitero P, Somerville J, Stone S, Aruta E, Spiegelhalter D, Rabajoli F (1994). Pregnancy in cyanotic congenital heart disease. Outcome of mother and fetus Circulation.

